# Preparative two-step purification of recombinant H1.0 linker histone and its domains

**DOI:** 10.1371/journal.pone.0189040

**Published:** 2017-12-05

**Authors:** Nives Ivic, Silvija Bilokapic, Mario Halic

**Affiliations:** Department of Biochemistry, Gene Center, University of Munich LMU, Munich, Germany; University of Lincoln, UNITED KINGDOM

## Abstract

H1 linker histones are small basic proteins that have a key role in the formation and maintenance of higher-order chromatin structures. Additionally, many examples have shown that linker histones play an important role in gene regulation, modulated by their various subtypes and posttranslational modifications. Obtaining high amounts of very pure linker histones, especially for efficient antibody production, remains a demanding and challenging procedure. Here we present an easy and fast method to purify human linker histone H1.0 overexpressed in *Escherichia coli*, as well as its domains: N-terminal/globular domain and C-terminal intrinsically disordered domain. This purification protocol relies on a simple affinity chromatography step followed by cation exchange due to the highly basic properties of histone proteins. Therefore, this protocol can also be applied to other linker histones. Highly pure proteins in amounts sufficient for most biochemical experiments can be obtained. The functional quality of purified H1.0 histone and its domains has been confirmed by pull-down, gel-mobility shift assays and the nuclear import assay.

## Introduction

The eukaryotic genome is packed into a dynamic macromolecular complex called chromatin. While this packing is necessary to arrange the genome within the nucleus, it prevents accessibility of DNA for replication, transcription and repair. To allow for these processes to happen the chromatin has to be rearranged by an interplay of different factors, such as chromatin remodellers, histone modification enzymes and histone chaperones [1,2].

The basic repeating unit of chromatin is the nucleosome, a DNA-protein complex. Two copies of H2A, H2B, H3 and H4 histones build a histone octamer, around which 145–147 base pairs (bp) of DNA are wrapped and completes a nucleosome core particle (NCP) [3]. The NCPs are connected by a short stretch of linker DNA. The linker histone H1 binds to the entry/exit sites of DNA on the NCP and interacts with the linker DNA [4,5]. NCP with the linker DNA and the histone H1 builds the nucleosome.

The core histones, H2A, H2B, H3 and H4, are relatively small proteins (10 to 15 kDa) characterized by a central α-helical globular domain, a histone-fold motif. Histone-folds are flanked by N- and C-terminal extensions, called the histone tails [2]. Histone H1 is larger than the core histones (~20 kDa). It has a short, unstructured N-terminus that is subject to post-translational modifications (PTMs) [6]. A globular ~80 amino acid central domain contains a winged helix motif and is sufficient for specific binding to the nucleosome *in vitro* [7]. An extended, positively charged C-terminal tail of ~100 amino acids plays the main role in condensing the nucleosomal fibre [8,9]. Interaction with the nucleosome induces shortening of the histone H1 C-terminal tail, resulting in a folded state [10].

One copy of H1 is found per nucleosome, while two copies of each of the core histones are required. Biochemical and biophysical analyses have demonstrated two different modes of H1 binding to nucleosomes [11,12]. In ‘on-dyad mode’, the globular domain of H1 binds symmetrically at the nucleosome dyad and interacts with similar lengths of linker DNAs entering and exiting the particle. In an ‘asymmetric mode’, the domain binds asymmetrically and interacts with different amounts of linker DNA [4,5,13]. The existence of alternative binding configurations reflects the conformational dynamics of linker histones *in vivo* [14]. Histone H1 is not as strongly incorporated into the chromatin as the core histones. An average residence time of H1 on a nucleosome is about a minute [15,16]. Thus, the vast majority of H1 molecules are bound to chromatin but the individual molecules are rapidly exchanging between chromosomal locations.

Linker histones are unequally distributed within the cell, as higher levels of H1 are detected in condensed heterochromatic regions than in euchromatic regions [17]. Additionally, H1 histone is much less evolutionarily conserved than H2A, H2B, H3 and H4 histones [18]. Higher eukaryotes contain multiple H1 subtypes. Eleven subtypes have been identified in humans [19]. The primary sequence conservation between the same H1 subtypes across different species is higher than a sequence conservation within subtypes from the same species. This evolutionary effort to conserve the sequence of each H1 subtype strongly points towards their specific functions [20,21].

Core and linker histone proteins are subject to a vast array of PTMs. Advances in mass spectrometry have revealed a large number of novel H1 subtype specific modifications [22]. Some modifications are specific to a given H1 variant, as the involved residue is not conserved among variants. Thus, they could play a role in interactions with specific protein partners and could contribute to variant-specific histone H1 functions [23].

The existence of multiple H1 subtypes and different posttranslational modifications make studying this protein family challenging. *In vivo* studies are hampered as it is difficult to obtain antibodies of high affinity and specificity for H1 variants, due to the protein family’s high heterogeneity. Previous methods of the linker histones purification rely on the histone solubilisation from the inclusion bodies using denaturants [24] or purification of the soluble histone using different ion exchange columns [25–27]. Here we describe a simple and efficient method for the rapid purification of human H1.0 subtype and its domains. The method can be applied to all linker histone subtypes as it relies on his-tag affinity chromatography and the high positive charge of H1.0 histone, a characteristic common to all linker histones. High amounts of the linker histones and its domains, easily obtained with our method, are needed for *in vitro* studies and production of specific antibodies.

## Materials and methods

### Plasmid construction

**Fig 1:**
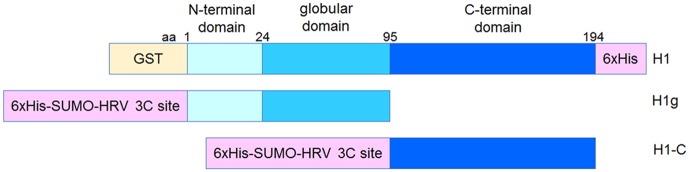
Domain architecture of recombinant *H*. *sapiens* H1.0 linker histone constructs. Full-length H1.0 was cloned into the pGEX-4T-1 vector with a GST fusion at the N-terminus and 6xHis-tag at the C-terminus. Globular H1.0 (H1g) and C-terminal H1.0 (H1-C) were generated by iPCR from full-length H1.0 with an N-terminal 6xHis-SUMO-HRV 3C site fusion in the pETDuet-1 vector.

The gene coding for *Homo sapiens* H1.0 was cloned using the sequence and ligation-independent cloning (SLIC) method into an in-house modified version of the pGEX-4T-1 bacterial expression plasmid, to yield an N-terminally GST-tagged protein followed by a human rhinovirus 3C (HRV 3C) protease recognition site [28]. A 6xHis-tag was subsequently added to the C-terminus of the H1.0 gene using iPCR [29]. In house modified pETDuet 1 plasmid containing a 6xHis-SUMO-tag followed by a HRV 3C cleavage site and *H*. *sapiens* H1.0 gene was further modified by iPCR to code for the N-terminal and globular domains of histone H1.0 (residues 1–95), or to code only for the C-terminal domain (residues 96-end) (Fig 1). Primers used for cloning by SLIC and iPCR are listed in the Table 1. All obtained plasmid sequences were checked by DNA sequencing (GATC Biotech AG, Konstanz, Germany). The resulting vectors were transformed into *Escherichia coli* Rosetta (DE3) strains (Novagen) for protein expression.

**Table 1:** List of primers used in this study.

	Cloning	Name	Sequence
**Cloning *H*. *sapiens* H1.0 gene into pGEX-4T-1**	SLIC	hs_h1_f	AGTTCTGTTCCAGGGGCCCGGATCCATGACCGAGAATTCCACGTC
	SLIC	hs_h1_r	CAGCGGTTTCTTTACCAGACTCGAGTCACTTCTTCTTGCCGGC
**Adding C-terminal 6xHis-SUMO to H1.0**	iPCR	hsh1_hisc_f	CATCACCACTGACTCGAGTCTGGTAAAGAA
	iPCR	hsh1_hisc_r	ATGGTGATGCTTCTTCTTGCCGGCC
**6xHis-SUMO-globular H1.0 domain into pETDuet-1**	iPCR	hsh1g_f	CCTAGGCTGCTGCCACC
	iPCR	hsh1g_r	TTAGGCTAGCCGGAAGGACCCCG
**6xHis-SUMO C-terminal H1.0 into pETDuet-1**	iPCR	hsh1C_f	TTAGGCTAGCCGGAAGGACCCCG
	iPCR	hsh1C_r	GGATCCGGGCCCCTGGAA

### Sequence and ligation-independent cloning (SLIC)

Plasmid vector (2 μg) was digested with 1 U of BamHI-HF and XhoI restriction enzymes (NEB) at 37°C in CutSmart buffer for 2.5 h. After digestion, the vector was gel purified and extracted using the NucleoSpin Gel and PCR Clean-up kit (Macherey-Nagel). *H*. *sapiens* H1.0 gene was amplified by polymerase chain reaction (PCR) using cMarathon DNA as a template. PCR product was purified using the NucleoSpin Gel and PCR Clean-up kit (Macherey-Nagel). One microgram of digested plasmid and 1 μg of the insert were treated separately with 0.5 U of T4 DNA polymerase in T4 DNA ligase buffer (NEB) in a 20 μl reaction at 22°C for 30 min. The reaction was stopped by adding 1/10 of the volume of 10 mM dNTP mix. 10 μl annealing reactions were set up using 1:1, 1:3 and 1:5 insert to vector ratios, with 150 ng of the vector, 1x T4 DNA ligation buffer (NEB), an appropriate amount of insert, and water. Reactions were incubated at 37°C for 30 minutes [28]. 5 μl was used to transform 50 μl of competent XL1-Blue *E*. *coli* cells.

### Inverse PCR

iPCR reactions were set up in a total volume of 15 μl. Products were purified using the NucleoSpin Gel and PCR Clean-up kit (Macherey-Nagel). 10 μl of purified PCR product was incubated in a total volume of 20 μl with 1× T4 DNA ligase buffer and 5 U of T4 PNK for 1 h at 37°C. T4 DNA ligase (200 U) was added to the reaction and incubated for 1 h at room temperature. Finally, the reaction was incubated for 1 hour at 37°C after addition of 10 U of DpnI. 5 μl was used to transform 50 μl of competent XL1-Blue *E*. *coli* cells [29].

### Protein expression and purification

Transformed *E*. *coli* Rosetta cells were grown in LB medium containing appropriate antibiotics. The culture was grown at 37°C to an optical density of ~0.6 at 600 nm, shifted to 18°C for 30 min and induced with 0.4 mM isopropyl-thiogalactopyranoside. After an overnight induction at 18°C, cells were collected by centrifugation at 4000 *g* for 10 min at 4°C. Pelleted cells were re-suspended in chilled lysis buffer (50 mM sodium-phosphate, pH 8.0, 500 mM NaCl, 20 mM imidazole, 10% glycerol (v/v), 3 mM β-mercaptoethanol, 0.1 mM phenylmethanesulfonyl fluoride). A French press was used to disrupt the cells. Generated lysate was centrifuged at 17 000 *g* for 20 min and the supernatant incubated with Ni Sepharose 6 Fast Flow resin (GE Healthcare) for 30 min at 4°C. The resin was washed three times with 10 bed volumes of lysis buffer in batch and loaded onto a disposable column (Pierce). On the column, the resin was washed with 4 bed volumes of washing buffer (50 mM sodium-phosphate, pH 8.0, 500 mM NaCl, 40 mM imidazole, 3 mM β-mercaptoethanol, 0.1 mM phenylmethanesulfonyl fluoride) before the protein was eluted with 5 bed volumes of elution buffer (50 mM sodium-phosphate, pH 8.0, 500 mM NaCl, 300 mM imidazole, 10% glycerol (v/v), 3 mM β-mercaptoethanol, 0.1 mM phenylmethanesulfonyl fluoride). Prior to the elution, the full-length H1.0 histone was washed with the low-salt elution buffer (50 mM sodium-phosphate pH 8.0, 50 mM NaCl, 300 mM imidazole, 3 mM β-mercaptoethanol, 0.1 mM phenylmethanesulfonyl fluoride). This step does not elute the H1.0 histone, but removes additional impurities which drastically minimizes precipitation of H1.0 in the subsequent purification steps. By contrast, H1.0 histone domains elute in the low-salt elution buffer; therefore, this washing step was not used for their purification.

HRV 3C protease was added to the eluted H1.0 histone protein or H1.0 globular domain in 1:100 mass ratio and the samples were dialyzed overnight against 15 mM HEPES/NaOH, pH 7.5, 300 mM NaCl, 10% glycerol (v/v), 1 mM EDTA and 1 mM DTT at 4°C. To avoid partial precipitation of the full-length H1 histone during overnight dialysis step, NaCl concentration was kept at 300 mM.

Due to its high positive charge, full-length H1.0 histone or H1.0 histone globular domain were further purified via cation-exchange chromatography (HiTrap SP FF column). To facilitate binding onto the column, the sample was diluted with buffer containing only 15 mM HEPES/NaOH, pH 7.5 and 10% glycerol (v/v) to reduce salt concentration in the sample to 150 mM before loading onto the column. A linear 0.15–2 M NaCl gradient in elution buffer was passed through the cation-exchange column. Fractions with the protein were analysed on a SDS-PAGE gel. Histone H1.0 protein and its globular domain were concentrated after dialysis (15 mM HEPES/NaOH, pH 7.5, 350 mM NaCl, 1 mM DTT) using Amicon Ultra-15 centrifugal filter (10 kDa cut off). For long-term storage, the concentrated protein was aliquoted, snap frozen in liquid N_2_ and stored at -80°C.

After elution from Ni Sepharose resin, H1.0 C-terminus was mixed with HRV 3C protease (~ 1:100 mass ratio) and dialysed against 15 mM HEPES/NaOH pH 7.5, 150 mM NaCl, 1 mM EDTA and 1 mM DTT. During the cleavage, H1.0 C-terminus precipitated due to its high positive charge, while 6xHis-SUMO tag remained soluble. Similar behaviour is also observed for core histones when they are dialysed in the buffer with low salt. H1.0 C-terminus was spun down (10 min, 4000 *g*, 4°C) and then dissolved in buffer containing 15 mM HEPES/NaOH pH 7.5, 2 M NaCl and 1 mM DTT. The protein was concentrated using 3 kDa cut-off membrane (Amicon Ultra).

*S*. *pombe* Nap1 histone chaperone was purified as described in Ivic *et al*. [30]. Briefly, after the cell disruption with French press and clarification by centrifugation, the lysate was mixed with pre-equilibrated Ni Sepharose resin and incubated for 20 min at 4°C in the same buffer as was described for H1.0 protein. After binding, the resin was washed three times with 10 bed volumes of lysis buffer in batch and loaded onto a disposable column (Pierce). On the column, the resin was washed with 4 bed volumes of washing buffer (50 mM sodium-phosphate, pH 8.0, 500 mM NaCl, 40 mM imidazole, 3 mM β-mercaptoethanol, 0.1 mM phenylmethanesulfonyl fluoride). Nap1 was eluted with 5 bed volumes of elution buffer (50 mM sodium-phosphate, pH 8.0, 500 mM NaCl, 300 mM imidazole, 10% glycerol (v/v), 3 mM β-mercaptoethanol, 0.1 mM phenylmethanesulfonyl fluoride). Protein was dialyzed overnight at 4°C against 20 mM HEPES/KOH, pH 7.5, 150 mM NaCl, 1 mM EDTA and 1 mM DTT and concentrated using 30 kDa cut-off membrane (Amicon Ultra).

### Size exclusion chromatography

Histone H1.0 protein and its domains were loaded onto a size exclusion Superdex 75 10/300 column (GE Healthcare) equilibrated in buffer (15 mM HEPES/NaOH, pH 7.5, 2 M NaCl, 1 mM DTT). Fractions containing protein were analysed on the 15% SDS-PAGE gel.

### Nucleosome reconstitution

DNA for nucleosome reconstitution was produced by PCR of a plasmid containing a Widom 601 DNA sequence [31] and an additional 40 bp on each side. PCR product was purified by phenol chloroform extraction. After ethanol precipitation, DNA was resuspended in buffer containing 2 M NaCl (25 mM Tris/HCl, pH7.5, 2 M NaCl, 1 mM DTT). Histone octamers were assembled from co-expressed and co-purified H2AH2B and H3H4 histone pairs, as described in Ivic et al. [30]. The octamers were separated from the excess H2AH2B dimer on the size exclusion chromatography column, equilibrated in 25 mM Tris/HCl, pH 7.5, 2 M NaCl, 1 mM DTT.

Nucleosome assembly was performed by mixing equimolar amounts of histone octamers and DNA. The reactions were placed into a dialysis button made from the lid of an Eppendorf tube and the reconstitution was done by the ‘double bag’ dialysis method as described in Ivic et al. [30].

### Gel mobility shift assay

Nap1 chaperone (1 μg) was mixed with full-length linker histone and its domains and incubated for 30 min at room temperature. H2AH2B histone dimer was used as a control for histone chaperone binding. The final concentration of the buffer was adjusted to 15 mM HEPES/NaOH pH 7.5, 150 mM NaCl and 1 mM DTT upon mixing all components. The samples were loaded on a 6% native polyacrylamide gel, run in 1x TBE on 120 V at 4°C and stained with SimplyBlue SafeStain.

A 227 bp DNA sequence based on the ‘601’ nucleosome positioning sequence with overhangs on both sides was PCR amplified, and purified using phenol-chlorophorm and ethanol precipitation. 0.5 μg of DNA was diluted in 15 mM HEPES/NaOH, pH 7.5, 150 mM NaCl, 1 mM DTT and mixed with purified H1 domains, while H2AH2B dimer was used as a control. After 30 min incubation on ice, the reaction mixtures were analysed on 6% native PAGE run in 1x TBE and stained with Sybr Gold.

### Pull-down

Five micrograms of GST-tagged histone H1.0 was bound to 15 μl of GST resin. The resin was washed two times with 20 mM HEPES/NaOH, pH 7.5, 150 mM NaCl and 1 mM DTT. The same amount of NCP was added to GST resin preincubated with H1.0 histone or buffer alone. NCP binding to the resin was done at 4°C with constant shaking for 30 min. Unbound protein was removed, the resin was washed two times with the same buffer as above, and protein was eluted with buffer supplemented with 15 mM glutathione. Samples were analysed on 17% SDS-PAGE and stained with SimplyBlue SafeStain.

### Nuclear protein import assay

Purified human H1.0 was labelled with NT-495 fluorescent dye using MO-L003 Monolith^™^ Protein Labeling Kit BLUE-NHS (Amine Reactive), NanoTemper Technologies. The nuclear import assay was performed according to the published protocol with some minor adjustments [32]. HeLa S3 cells were grown in 16 well plates in DMEM medium with 10% (v/v) fetal bovine serum and 1% (w/v) penicillin/streptomycin to 80% confluence. The media was removed and washed three times with 200 μl of cold transport buffer (TB) containing 20 mM HEPES/NaOH pH 7.4, 110 mM potassium acetate, 2 mM magnesium acetate, 1 mM EGTA, 2 mM DTT, 0.1 mM PMSF and 1 μg/ml each of leupeptin, pepstatin and aprotinine. Cells were permeabilazed with 0.005% digitonin for 5 min and washed again three times with TB. 1 ml of fresh cold TB or WGA was added and incubated for 15 min at room temperature. To remove all the cytosol, cells were washed another five times with 1 ml of TB. After washing 50 μl of the import reaction mix (0.4 μM ND-495 H1.0, 0.3 μM Impβ, 0.3 μM Imp7, 1 μM NTF2, 1 mM ATP, 0.1 mM GTP, 1 mg/ml CP, 15 U/ml CPK and if used 0.8 mg/ml import inhibitor wheat germ agglutinin (WGA)) was added to the cells and incubated for 30 min at room temperature. As negative controls, reaction mixtures without importin receptors were used. WGA blocks nuclear pores and was used as an additional negative control to confirm that the passage occurs only through the nuclear pore complex. After incubation, cells were again washed three times with 200 μl TB, stained with DAPI (300 nM) and then inspected under the fluorescent microscope. The experiment was repeated three times.

## Results and discussion

### Expression and purification of recombinant histone H1.0 protein and its domains

**Fig 2:**
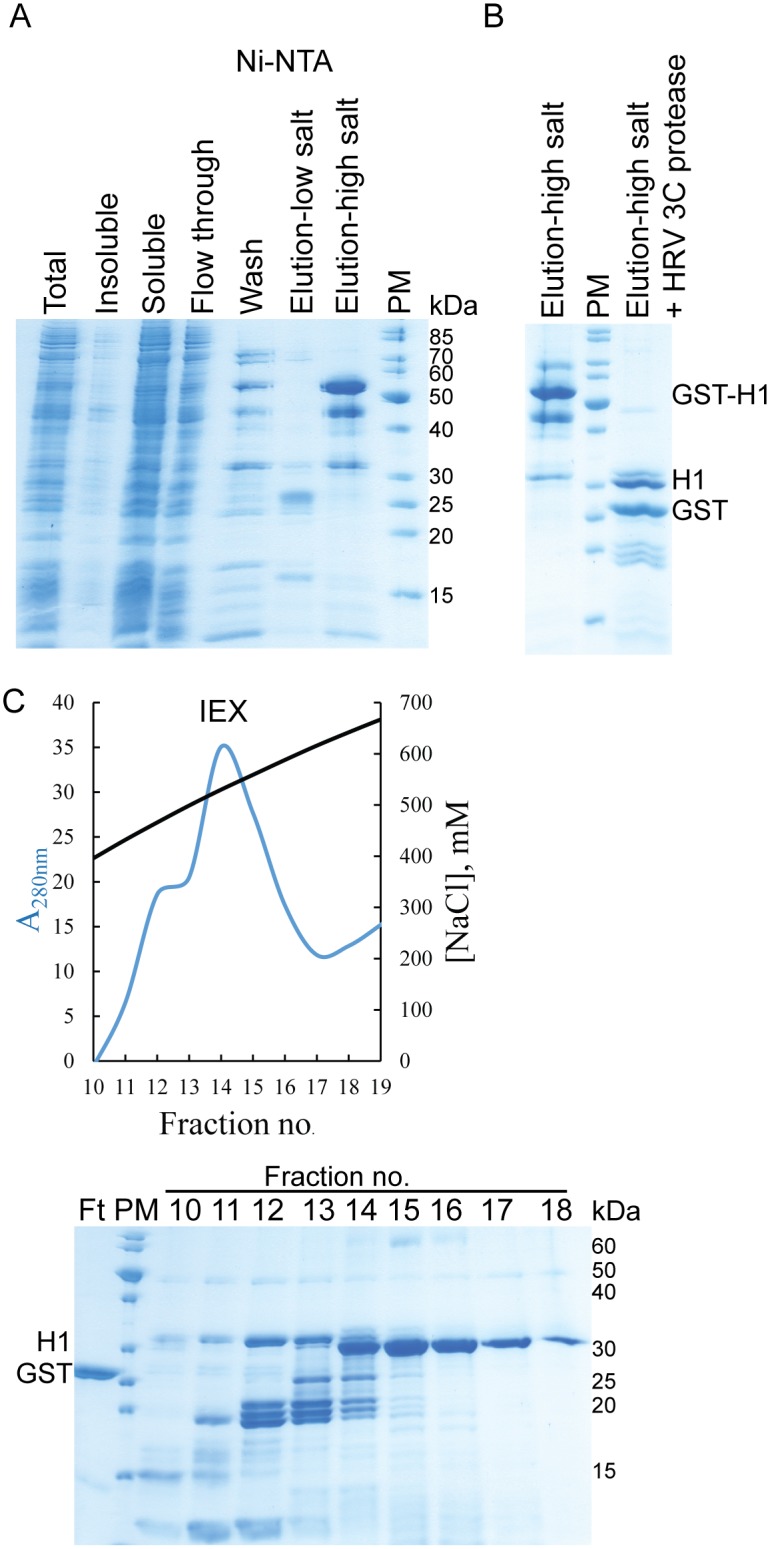
Purification of *H*. *sapiens* H1.0 linker histone. (A) SDS-PAGE analysis of expression and Ni-NTA purification of GST-H1.0-His6 (~55 kDa). (B) SDS-PAGE analysis of GST-H1.0-His6 before and after cleavage treatment with HRV 3C protease. Two prominent lower molecular bands can be observed, GST (~26 kDa) and H1.0-His6 (runs ~32 kDa). (C) Chromatogram and SDS-PAGE gel analysis of cation exchange chromatography. Ft, unbound fraction containing GST; PM, protein marker PageRuler Unstained Protein Ladder; 10–18, protein fractions containing full-length H1.

Establishing expression and purification of the core histones in *E*. *coli* cells has revolutionized the chromatin field. This approach proved to be very successful and resulted in the crystal structure of the nucleosome core particle [3,33]. In contrast, expression of linker histones in *E*. *coli* cells using pET vectors proved to be problematic [25,34]. We wanted to take an advantage of the T7 promoter, which is usually used for a high-level expression of proteins in bacteria, and reasoned that adding a bigger fusion tag N-terminally to histone H1 could enhance expression and solubility. The gene coding for *H*. *sapiens* histone H1.0 was cloned into two in-house modified bacterial expression plasmids: the pETDuet-1 vector containing an N-terminally 6xHis-SUMO-tagged protein followed by a HRV 3C protease recognition site, and the pGEX vector with a HRV 3C site following a GST domain. Recombinant histone H1.0 fusion proteins were successfully expressed in *E*. *coli* when induced overnight at 18°C. Both fusion proteins were soluble, although when using a GST-tag we obtained higher amounts (data not shown). Therefore, we focused on the N-terminally GST-tagged histone H1.0 construct. The final vector expressing the *H*. *sapiens* H1.0 histone variant contains an N-terminal GST-tag followed by a HRV 3C recognition site and a C-terminal 6xHis-tag (Fig 1). On SDS gels a predominant band corresponded to the full-length GST-tagged H1.0 with a few lower migrating bands corresponding to C-terminal protein truncations (Fig 2A and 2B, lanes labelled as Elution) (the same was observed for 6xHis-SUMO-tagged fusion protein, data not shown). To purify away degradation products, we added a C-terminal 6xHis-tag using iPCR [29]. The final vector expressing the *H*. *sapiens* H1.0 histone variant contains an N-terminal GST-tag followed by a HRV 3C recognition site and a C-terminal 6xHis-tag (Fig 1).

Following induction, histone variant H1.0 was purified by Ni-NTA affinity chromatography under native conditions (Fig 2A). Due to a high percentage of positively charged residues, buffers used for the purification contained 0.5 M salt to avoid protein precipitation. Upon purification, salt concentrations could be lowered to 0.3 M to keep H1.0 soluble even when the protein was highly concentrated. As with core histones, H1.0 linker histone is a highly basic protein and we used this property for its further purification. Upon elution from Ni-NTA resin, H1.0 histone was dialysed into buffer with lower salt and at the same time the GST-tag was proteolytically removed (Fig 2B). The dialysed sample was loaded onto a HiTrap SP column and subjected to a salt gradient. Cleaved GST-tag and HRV 3C protease did not bind to the column and eluted during sample loading (Fig 2C, lane Ft). Full-length H1.0 and its degradation products bind to the column due to high positive charge, but the full-length elutes significantly later allowing for the purification of clean full-length linker histone (Fig 2C, lanes 15–18).

In our experimental set-up, the GST domain served as a solubility tag and linker histone was purified using an incorporated C-terminal 6xHis-tag and the high intrinsic positive charge of the protein itself. Thus, the GST-tag can even be left on the linker histone during the purification and be used in the subsequent experiments, e.g. in the pull-down (see later).

**Fig 3:**
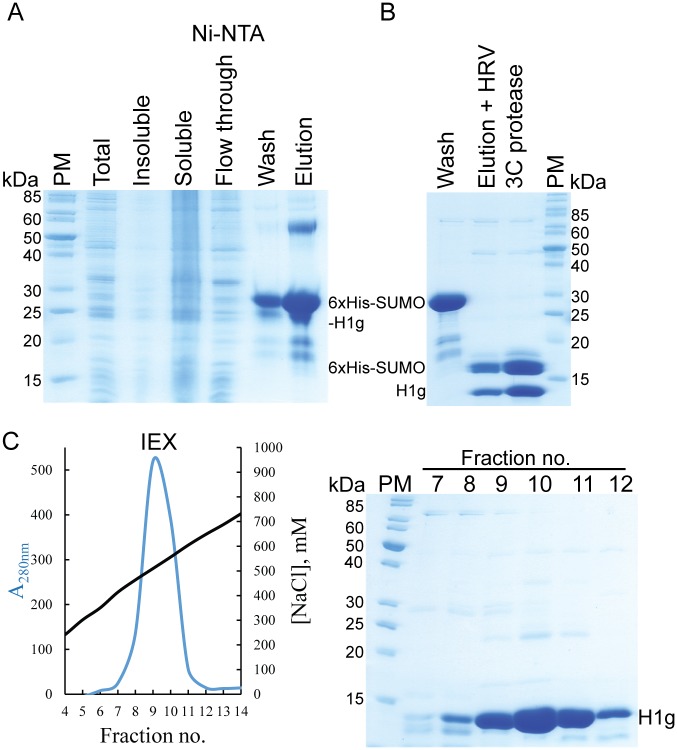
Purification of *H*. *sapiens* H1.0 linker histone globular domain. (A) SDS-PAGE analysis of expression and Ni-NTA purification of His_6_-SUMO-H1g. (B) SDS-PAGE analysis of His6-SUMO-H1g after cleavage treatment with HRV 3C protease. Two prominent lower molecular bands can be observed, His6-SUMO (~16 kDa) and H1.0g (~13 kDa). (C) Chromatogram and SDS-PAGE gel analysis of peak fractions (lanes 7–12) after anion exchange chromatography. H1g elutes with high ionic strength buffer (~500 mM NaCl). PM is protein marker PageRuler Unstained Protein Ladder, Thermo Scientific.

**Fig 4:**
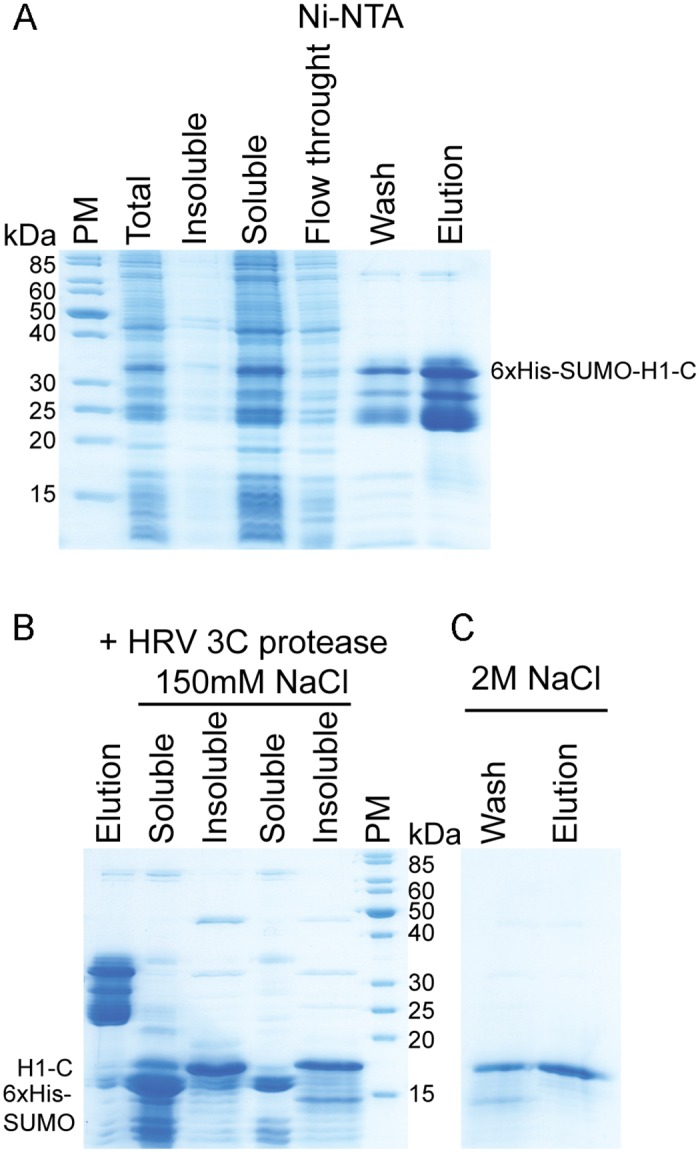
Purification of *H*. *sapiens* H1.0 linker histone C-terminal domain (H1-C). (A) SDS-PAGE analysis of expression and Ni-NTA purification of His_6_-SUMO-H1-C. (B) SDS-PAGE analysis of His_6_-SUMO-H1-C after cleavage treatment with HRV 3C protease. During dialysis in buffer containing 150 mM NaCl, the majority of H1.0 C-terminal domain precipitates (insoluble), while His_6_-SUMO remains soluble. (C) SDS-PAGE gel analysis of H1-C solubilized in 2 M NaCl. Protein marker (PM) is PageRuler Unstained Protein Ladder, Thermo Scientific.

Using iPCR [29] we have modified the full-length linker histone gene to code for the N-terminal globular domain (residues 1–95) or the C-terminal disordered domain of the linker histone (residues 96-end) (Fig 1). After the IMAC step, H1.0 histone globular domain was further purified via cation-exchange chromatography, using the same experimental setup as for the full-length protein (Fig 3A–3C). The C-terminal domain of linker histone is highly charged. After the initial purification using the 6xHis-tag (Fig 4A), this domain was dialysed in buffer with a physiological salt concentration where it precipitates (Fig 4B). We used this as a final purification step to remove soluble contaminants (Fig 4B and 4C). This was expected, as core histones also precipitate in buffer with physiological salt concentration due to their charge [35].

**Fig 5:**
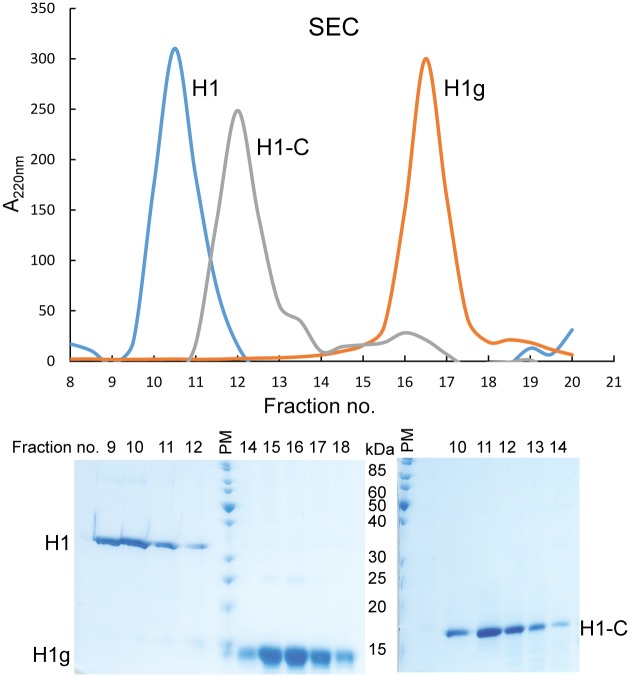
Gel filtration analysis of purified full-length H1.0 linker histone and its domains H1g and H1-C. Peak fractions were analysed by SDS-PAGE. PM, protein marker PageRuler Unstained Protein Ladder, Thermo Scientific.

We noticed that the full-length H1.0 migrates on SDS-PAGE as a significantly larger protein than its true molecular weight (Fig 2C). It appears as ~32 kDa protein on the denaturating gel although its molecular weight is 20 kDa. The N-terminal globular domain on the SDS-PAGE runs as a slightly larger protein than the apparent mass (13 kDa on SDS-PAGE, 10 kDa the molecular weight) (Fig 3C). However, the C-terminal domain migrates at a significantly larger apparent molecular weight (18 kDa on SDS-PAGE, 10 kDa the molecular weight) (Fig 4B). The highly charged histone tails are causing this anomalous behaviour. We also noticed similar anomalous behaviour for full-length linker histone and its C-terminal domain on size exclusion chromatography (Fig 5). While the globular domain runs with respect to its molecular weight, the disordered C-terminal domain and thus also the full-length protein migrate as significantly bigger proteins. This is due to their larger radius of gyration which is a consequence of their disordered nature.

While the yield of purified full length H1.0 is around 1 mg per litre of bacterial cell culture, amounts of purified globular and C-terminal H1.0 domains are significantly higher, around 5 mg/L. In two work days we purified soluble linker histone and its domains thus avoiding laborious and lengthy denaturation and refolding steps. In the recent years, several different approaches have also been established for the core histones which avoided purification from inclusion bodies [30,36–38].

### Testing the functionality of full-length linker histone and its domains

**Fig 6:**
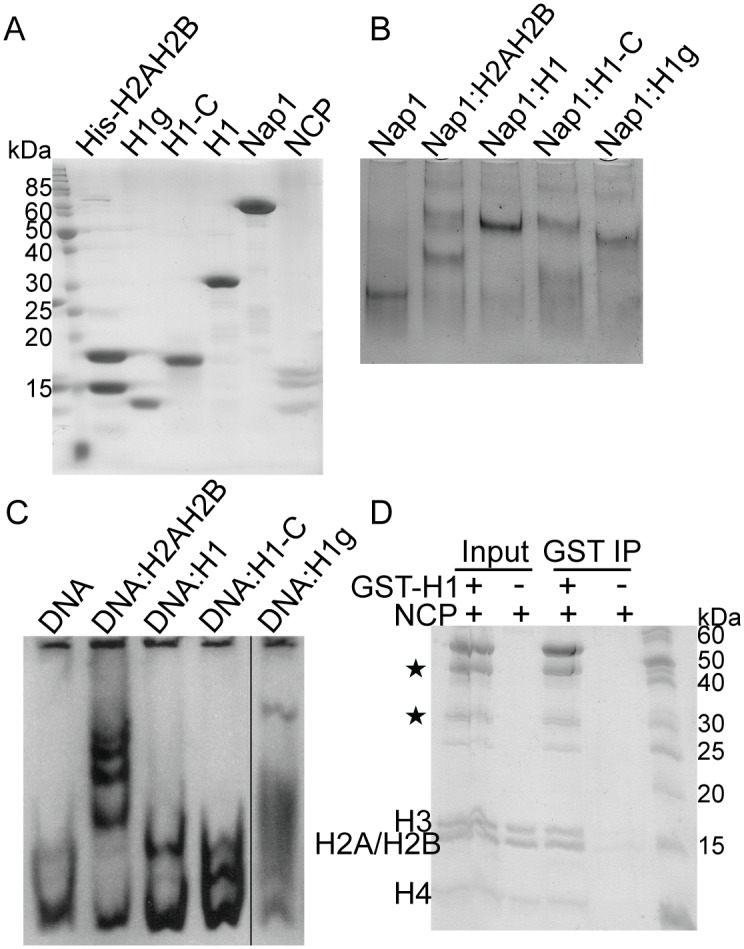
Purified recombinant H1.0 and its domains are functional. (A) SDS—PAGE analysis of purified proteins used for *in vitro* assays. Protein samples were analysed using 17% SDS—PAGE gels. Proteins were visualized by staining with SimplyBlue SafeStain. PageRuler Unstained Protein Ladder, Thermo Scientific, was used as protein marker. (B) Native PAGE of complex formation between histone chaperone Nap1 and H1 histone or its domains. H2AH2B histone dimer was used as a control for Nap1 binding. A histone chaperone:histone complex migrates slower compared to the histone chaperone alone. Several slower migrating bands can be observed in native polyacrylamide gels, indicative of the formation of higher order association states, typical for Nap1 chaperone. (C) Native PAGE of complex formation between DNA and H1 histone or its domains. H2AH2B histone dimer was used as a control for DNA binding. DNA:histone complex migrates slower compared to DNA. The complexes were visualized by SyberGold. (D) GST pull-down assays using GST-H1.0 and NCP. The proteins were visualized by Simply Blue SafeStain.

To keep histones soluble for incorporation into chromatin and to prevent nonspecific interactions with other negatively charged molecules in the cell, they are bound by acidic proteins termed histone chaperones [39]. One such chaperone is nucleosome assembly protein 1 (Nap1) [39,40]. To determine the functionality and quality of our protein preparations we tested their binding to *S*. *pombe* Nap1. Nap1 chaperone was incubated with full-length histone H1 or its domains, and complex formation was assessed by electrophoretic gel mobility shift analysis (Fig 6B). Our data show that Nap1 binds not only the full-length linker histone, but also both of its domains. H2AH2B histone dimer was used as a positive control for histone chaperone binding. For all complexes we observed several bands in native polyacrylamide gels, indicative of the formation of higher order association states. This is in agreement with the known oligomerization features of Nap1 chaperone [40].

**Fig 7:**
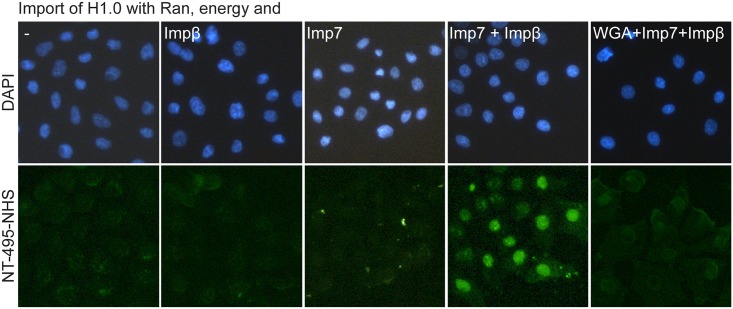
Nuclear import assay of recombinant H1.0 in digitonin-permeabilized HeLa cells reconstituted with the import factors. H1.0 import is dependent on the presence of both Importin 7 and Importin β. Linker histone import is blocked if WGA is added to the reaction. Histone H1.0 alone or with only one importin were used as a negative control. On the upper panels, cell nuclei stained with DAPI are shown. On the lower panels, cells with NT-495 labelled H1 are shown.

H1 histone binds free DNA, although it prefers nucleosomes. Purified H1 and its domains were tested for DNA binding [41]. Purified proteins were able to shift free DNA, similarly to H2AH2B dimer that was used as a positive control (Fig 6C). Finally, histone H1 was also tested for nucleosome binding (Fig 6D). DNA longer than 145–147 bp was chosen as H1 histone binds linker DNA in addition to the nucleosome core. SDS-PAGE analysis of the pull-down reveals NCP proteins (H3, H2A, H2B and H4) bound to GST resin only in the presence of GST-tagged H1 histone (Fig 6D). To address the possibility of non-specific binding of H1 to the chaperone or the DNA, we performed the nuclear import assay using purified and fluorescently labelled H1 histone. Our results confirm that nuclear import of the linker histone is possible only in the presence of importin 7/β heterodimer (Fig 7), indicating that the histone H1 purified by our method is functional [42].

The described protocol enables rapid purification of high amounts of soluble and pure linker histone H1.0 and its domains. Histones are among the most highly conserved proteins, with their biochemical properties remarkably similar across many species. Therefore, the described approach can be used for other linker histone subtypes as it takes advantage of the protein’s intrinsic high positive charge, and a very common His-affinity tag. Our approach is fast and efficient so that multiple samples, such as different linker histone isoforms, can be processed in parallel. In addition, numerous modifications of each linker histone gene, such as introducing mutations or designing different domains, can be quickly done using iPCR. The ability to obtain different highly purified linker histone subtypes is crucial for *in vitro* studies and to produce specific antibodies. We expect that our simple approach to obtain highly purified linker histone subtypes will contribute to the understanding of their specific roles in the chromatin.
